# GARP: a surface molecule of regulatory T cells that is involved in the regulatory function and TGF-β releasing

**DOI:** 10.18632/oncotarget.8753

**Published:** 2016-04-15

**Authors:** Liping Sun, Hao Jin, Hui Li

**Affiliations:** ^1^ Department of Immunology, Tianjin Medical University Cancer Institute and Hospital, Tianjin, China; ^2^ Department of Gastrointestinal Cancer Biology, Tianjin Medical University Cancer Institute and Hospital, Tianjin, China; ^3^ Key Laboratory of Cancer Immunology and Biotherapy, Tianjin, China; ^4^ National Clinical Research Center of Cancer, Tianjin, China

**Keywords:** regulatory T cells, glycoprotein A repetitions predominant, transforming growth factor β

## Abstract

There are many molecules that define regulatory T cells (Tregs) phenotypically and functionally. Glycoprotein A repetitions predominant (GARP) is a transmembrane protein containing leucine rich repeats. Recently, GARP is found to express highly on the surface of activated Tregs. The combination of GARP and other surface molecules isolates Tregs with higher purity. Besides, GARP is a cell surface molecule of Tregs that maintains their regulatory function and homeosatsis. GARP has also been proved to promote the activation and secretion of transforming growth factor β (TGF-β). Moreover, its potential value in cancer immunotherapy is also discussed in this work.

## INTRODUCTION

CD4^+^ regulatory T cells (Tregs) are immunosuppressive T cells that play an important role in immune homeostasis. Because of difference in generation and mechanisms of action, there are two types of CD4^+^ Tregs: naturally occurring Tregs (nTregs) and induced Tregs (iTregs) [1, 2]. nTregs develop in thymus and migrate to peripheral tissues [1]. iTregs are derived from mature peripheral naive CD4^+^ T cells under a variety of conditions [3]. Although nTregs and iTregs differ in mechanisms of generation and functional properties, they share a similar phenotype [4].

A number of surface and intracellular molecules express highly in Tregs [1]. There are also some molecules that show low/negative expression on Tregs [5]. Because the use of them as reliable markers of Tregs is still limited, there are still many obstacles in therapeutic applications of Tregs. Glycoprotein A repetitions predominant (GARP) is a transmembrane protein consisting of 662 amino acids [6]. The expression of GARP is highly on the surface activated Tregs and increases the suppressive function of Tregs [7]. Additionally, GARP can bind to latent transforming growth factor β (TGF-β), thus promoting secretion and activation of TGF-β [8]. TGF-β plays a critical rule for homeostasis and function of Tregs [9]. Finally, we discuss potential therapeutic value of GARP in cancer immunotherapy.

## TREG MARKERS

To fully understand the role and function of Tregs, a number of surface and intracellular markers have been proved to be useful for identifying suppressive Tregs subsets, as summarized in Table 1. In 1970s, a subset of T cells with suppressive capacity was described firstly [10]. However, the lack of surface markers impedes the process of these cells. In 1995, Sakaguchi et al. first discovered that CD4^+^ T cells expressing CD25 were anergic and suppressive in mice [11]. Subsequently, CD25 is identified as a surface marker necessary for Tregs development and function [12, 13]. However, only 1~2% of CD4^+^ T cells with highest CD25 expression are Tregs with suppressive function in human peripheral blood [14, 15]. Thus, the use of CD25 as a surface marker of Tregs seems to be unconvinced, considering that human CD4^+^CD25^+^ T cells contain Tregs and activated Teffs [16]. Shortly thereafter, molecules like cytotoxic T lymphocyte-associated antigen 4 (CTLA-4) [17], inducible costimulator (ICOS) [18], tumor necrosis factor receptor 2 (TNFR2) [19], glucocorticoid-induced tumor necrosis factor receptor (GITR) [20], 4-1BB, OX40 [21], lymphocyte activation gene *3 (*LAG-3) [22] and latency associated peptide (LAP) [23] are reported to express on the surface of Tregs. Intracellular molecules Foxp3 [24] and Helios [25] also express in Tregs strongly and constitutively. Additionally, several surface molecules like CD127 [26], CD6 [27], CD26 [28] and CD49d [29] show low/negative expression on Tregs.

**Table 1:** Tregs markers

		Marker	Comment	Reference
Surface	High	CD25	Only 1~2% of CD4^+^ T cells with highest CD25 expression are Tregs with suppressive function in human peripheral blood	**[11, 14–16]**
		CTLA-4	The expression level of CTLA-4 is correlated with suppressive function of Tregs. CTLA-4 is also rapidly induced in Teffs upon activation	**[17, 41]**
		ICOS	The majority of mouse Tregs express ICOS. ICOS^+^ Tregs possess superior suppressive activity than ICOS^−^ Tregs in mice. Both human ICOS^+^ Tregs and ICOS^−^ Tregs have strong suppressive capacity	**[18, 42–44]**
		TNFR2	The majority of human and mice nTregs are TNFR2 expressing cells. Mouse TNFR2^+^ Tregs have the maximally suppressive function. Mouse TNFR2^−^ Tregs, even if they are FoxP3^+^ cells, only have minimal or no suppressive activity	**[19, 45, 46]**
		GITR	High surface expression of GITR is detected on Tregs. It is also detectable on Teffs and the function of Tregs are not dependent on GITR	**[20, 47, 48]**
		OX40	4-1BB and OX40 are preferentially up-regulated on the surface of Tregs by TNF stimulation and can promote the proliferation or survival of Tregs. However, OX40 and 4-1BB also inhibit the development and suppressive activity of Tregs	**[21, 49, 50]**
		4-1BB		
		LAG-3	LAG-3 defines an active Tregs subset and contributes to their function. All T cells upon activation express LAG-3	**[22, 51, 52]**
		LAP	LAP expression is high on the surface of activated Tregs and induces TGF-β secretion. LAP^+^ Tregs of lamina propria show reduced suppressive activity and increased IL-17 expression in active ulcerative colitis	**[23, 53]**
		GARP	GARP is highly expressed on activated Tregs and contributes to their suppressive activity	**[7]**
	Low/negative	CD127	Tregs are generally characterized by low surface expression of CD127. However, down-regulation of CD127 is also observed on activated Teffs. Activation of mouse Tregs also results in up-regulation of CD127 expression in vivo and vitro	**[26, 54]**
		CD6	Low/negative expression of CD6 is reported on human nTregs	**[27]**
		CD26	Human Tregs are characterized by low/negative CD26 expression	**[28]**
		CD49d	CD49 is present on proinflammatory effector cells but absent on Tregs. The combination of CD127 and CD49d identifies highly pure population of Tregs	**[29]**
Intracullular		Foxp3	Foxp3 is the most specific marker for Tregs, but Foxp3 expression is found in human activated Teffs. Foxp3 expression in Teffs confers neither a regulatory phenotype nor suppressor function	**[24, 31]**
		Helios	Helios is expressed exclusively in mouse nTregs but not in human nTregs	**[55]**

The transcription factor Foxp3 is to date the most specific marker for Tregs. It is necessary for the development and function of Tregs [30]. However, Foxp3 expression is not specifically in human Tregs and is found in activated Teffs [31]. Foxp3 expression in Teffs confers neither a regulatory phenotype nor suppressor function [31–34]. Besides, Foxp3 cannot be used to separate functional Tregs for further researches because of its intracellular location [35]. In brief, the use of Foxp3 as a reliable marker for bona fide human Tregs is limited and not adequate to discriminate suppressive Tregs from other T cells.

**Figure 1:**
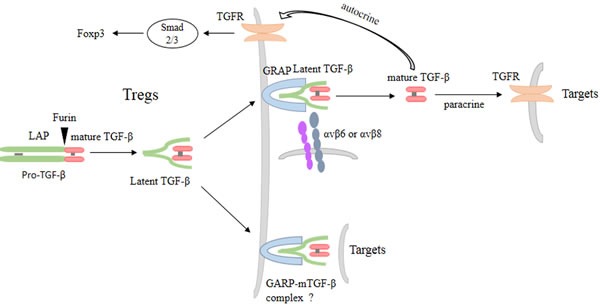
TGF-β and GARP maintain homeostasis and function of Tregs TGF-β is synthesized as a pro-TGF-β precursor, a homodimer that includes LAP and mature TGF-β. Then pro-TGF-β are cleaved by the enzyme furin to form latent TGF-β, where mature TGF-β remain noncovalently bind to the LAP. Membrane form of GARP transports and anchors latent TGF-β to the surface of Tregs. The release of mature TGF-β from the surface latent TGF-β/GARP complex can be mediated by integrin. Mature TGF-β in turn acts on Tregs themselves, forming a positive and autocrine TGF-β feedback loop. Mature TGF-β also acts on target cells in paracrine manner. Besides, the surface latent TGF-β/GARP complex can also directly act on target cells and increases Tregs suppressive function, but the mechanism is still unclear.

In recent years, GARP is discovered to express highly on the surface of activated human Tregs *in vitro* [36]. Freshly isolated human Tregs and Tregs clones express a low level of surface GARP. With T cell receptor (TCR) stimulation, the surface expression of GARP on Tregs and Tregs clones is rapidly upregulated [9, 36]. Although GARP mRNA is detectable in human Th clones even its level close to some Treg clones, GARP is not detected on the surface of stimulated Th clones. The expression of GARP is very low (~3%) on the surface of activated Teffs [9, 37]. In mice, GARP expression is also highly on the surface of activated Tregs [38]. Moreover, GARP expression is restricted to Foxp3^+^ population and more than 90% of the CD25^+^GARP^+^ T cells express Foxp3 [37]. Treg-specific demethylated region (TSDR) is an conserved non-coding region with CpG motifs in Foxp3 locus and unmethylated in Tregs to induce Foxp3 expression [39]. The portion of TSDR in CD4^+^GARP^+^ T cells is 74%, which is similar to the portion in CD4^+^CD25^+^CD127^low^ T cells (74±4%) and higher than in CD4^+^CD25^hi^ T cells (62 ±2%) [37]. A negligible expression (5±1%) of CD154, an activation marker for Teffs, is observed on the surface of Tregs [40]. The combination of CD154 and GARP isolates Tregs with the highest suppressive activity [36]. Thus, GARP may serve as an activated Tregs surface marker. The combination of GARP and other molecules can be used to separate functional Tregs.

## GARP GENE

Human GARP gene is firstly isolated in 11q13.5-11q14 chromosomal region in human breast carcinoma cells and defined as DI1S833E. The homologous sequence in mouse is located on Chromosome 7, region 7E-7F [56, 57]. GARP gene that consists of two coding exons is expressed at two major transcripts of 4.4 and 2.8 kilobases respectively. The signal peptide and nine amino acid residues are encoded by the first exon. The second and the large one contains a putative extracellular region which encodes twenty leucine rich repeats (LRRs). The large exon also encodes a putative transmembrane domain followed by a short intracellular region [6, 58]. GARP gene is expressed in various tissues including placenta, lung, kidney, heart, liver, skeletal muscle, pancreas and lymphoid tissues[58]. Additionally, GARP gene is detected in multiple cell types such as megakaryocytes, platelets, B lymphocytes, T lymphocytes, mesenchymal stromal cells (MSCs) and human umbilical vein endothelial cells [59, 60]. Interestingly, an amplification of GARP gene has been found in tumors, particularly in invasive, metastatic or treatment-resistant tumors [61–64]. These may suggesting a potential role of this gene in regulating the aggressive ability of tumor.

## GARP EXPRESSION

The extracellular portion of GARP is mostly composed of LRRs, thus GARP is also known as leucine rich repeats containing 32 (LRRC32). The structure of extracellular portion of GARP is similar to other members in LRR protein family, which play a role in protein-protein interactions and signal transduction [6, 65]. Protein structure prediction hints that the extracellular portion of GARP is high homology to the ectodomain of Toll-like receptor 3 (TLR-3), which is a horseshoe- shaped solenoid [66, 67]. GP96 serves as an essential chaperone for folding TLRs [68]. GP96 is also an essential chaperone for cell-surface GARP [69]. Similar to TLR3, three of five potential glycosylation sites of GARP are positioned on the concave face. The three potential glycosylation sites predict as potential ligand binding and oligomerization sites [70]. TLRs can recognize pathogen-associated molecular patterns and also bind with autologous molecules [71]. However, the possible ligands for GARP has not been reported.

GARP gene is detected in multiple cells types, however, only MSCs, hepatic stellate cells, platelets and Tregs are reported to express GARP on their membrane [6, 59, 72]. GARP expression has been proved to be regulated by microRNA because the distal part of 3′ untranslated region (UTR) contains five highly conserved sequence [76]. MiR-142-3p, miR-181a, miR-185, miR-24 and miR-335 are considered to bind to the 3′UTR of GARP to repress its expression [77–79]. Whereafter, miR-142-3p represses posttranscriptional regulation of GARP expression by argonaute 2-associated degradation of GARP mRNA [79]. Thus, downregulation of miRNA may be one way to induce GARP expression in Tregs.

## THE FUNCTION OF GARP IN TREGS

### GARP increases the suppressive function of tregs

The potential role of GARP in Tregs function has been analyzed in human Tregs. Compared with the GARP- Tregs, human GARP^+^ Tregs are more potent in inhibiting the proliferation of Teffs *in vitro*. GARP^+^ Tregs also secrete less IL-2 and IFN-γ [37, 67, 73]. GARP^+^ Tregs is able to secrete abundant TGF-β and IL-10 [74, 75]. In addition, GARP^+^CD154^−^ Tregs are functionally much better than CD4+CD25^high^ or CD4+CD25^high^CD127^low^ Tregs *in vitro* and in suppressing alloreactive immunoresponses in a humanized mouse model [36]. The suppressive function of Tregs are modestly and significantly impaired by GARP downregulation. The expression of CD27, CD83 and Foxp3 in Tregs are also significantly inhibited by GARP downregulation [67]. CD27 and CD83 contribute to the immunosuppressive function of Tregs by inducing Foxp3 expression [76, 77]. Tregs significantly suppress allergen-induced gut inflammation in a humanized mouse model. The suppressive effects of Tregs are further increased by activation before injection. Depletion of GARP-expressing cells among activated Tregs before injection and administration of GARP abrogate their inhibitory effects [78].

GARP overexpression endows non-Tregs with a partial or full regulatory phenotype and function. GARP overexpression in non-Tregs sustains the expression of Foxp3. GARP also increases the expression of other Treg-associated molecules including CD25, CTLA4, LGALS3, LGMN and CD27 in non-Tregs [7, 37]. In non-Tregs, GARP overexpression decreases the production of IL-2 and IFN-γ, and impairs the proliferative capacity. Furthermore, non-Tregs tranduced with GARP acquires suppressive activity. TCR stimulation further increases the surface expression of GARP on Tregs and intensifies effects of GARP on non-Tregs [67, 79]. Besides, genes representing a Treg-signature also upregulate in GARP transduction cells [67]. TGF-β-treated Teffs not express surface GARP upon TCR activation and is not suppressive [37]. These results suggest that GARP increases immunosuppressive function of Tregs, at least in part, but the mechanism of GARP-mediated inhibition of Tregs remains to be further studied.

### The relationship between GARP and Foxp3

The relationship between GARP and Foxp3 is still in debate. The expression of Foxp3 appears to be not required for the expression of GARP because the expression of GARP is completely normal in Foxp3 knockdown Tregs [8, 37]. Silencing GARP only attenuates Tregs suppressive activity, but not affect the expression of Foxp3. The suppressive function of GARP^+^ Tregs are not altered by silencing Foxp3 [7]. Furthermore, following TCR activation, GARP/LAP are up-regulated on CD4^+^Helios^+^ T cells regardless of Foxp3 expression [74, 80]. These indicates that the expression of GARP and Foxp3 are independent probably, but all the experiments are carried out *in vitro*. What is the real situation or relationship between GARP and Foxp3 *in vivo*? It remains elusive and needs further studies to find out the truth.

However, controversy is always there. GARP expression is restricted to Foxp3+ population [40]. Foxp3 knockdown decreases the expression of GARP and suppressive function of Tregs [67]. Moreover, the induction of Foxp3 expression in Tregs precursors is accompanied by GARP [81, 82]. This provides compelling evidence that the induction of GARP in Tregs is dependent on Foxp3 expression and GARP can also induce Foxp3 expression. We believe that there must be links between GARP and Foxp3 in the complex environment of human body.

### GARP is involved in the function and homeostasis of tregs *via* TGF-β

#### GARP promotes TGF-β secretion and activation

The secretion and activation of TGF-β is a multi-step process. TGF-β is firstly synthesized as pro-TGF-β precursor which exists as a homodimer in cells. Then, the homodimerized pro-TGF-β is proteolytically cleaved by furin, resulting in the product called latent TGF-β. In latent TGF-β, LAP noncovalently binds to mature TGF-β and prevents mature TGF-β from binding to its receptor [9]. The release of mature TGF-β from latent TGF-β complex needs three steps [83]: 1) Disulfide linkage between latent TGF-β to latent TGF-β binding proteins (LTBPs). Two same cysteines, Cys-4 of the latent TGF-β dimmer, can link to the cysteines of LTBPs through disulfide bond [84]. 2) Combination between integrin and latent TGF-β. The structure of latent TGF-β is reported in 2011. The whole structure is a ring-like shape and mature TGF-β is in the centre of the ring [85]. Cys-4 located at the bottom of the ring would be linked to LTBPs. The RGD motifs located to each shoulder of the ring can be recognized by integrin expressed on cell surface [86]. 3) Contractile force. Since latent TGF-β and LTBPs are fixed on cell surface and extracellular matrix cytoskeleton respectively, the contractile force is able to release mature TGF-β from latent TGF-β [87]. However, contractile force is not always required for TGF-β activation because the metalloprotease 14 has been shown to cleave latent TGF-β without contractile force [88].

It has been proved that GARP is essential for the surface expression of latent TGF-β on Tregs. GARP expressed on surface of Tregs possesses similar role as LTBPs. GARP directly binds to latent TGF-β through disulfide linkage and noncovalent association [73]. The latent TGF-β is anchored on Tregs membrane, so the GARP associated TGF-β can describe as membrane bound TGF-β (mTGF-β). Cys-192 and Cys-331 of GARP disufide link to Cys-4 of latent TGF-β. Though Cys-192 and Cys-331 mutate to alanine, GARP can still noncovalently associate with latent TGF-β to prevent TGF-β secretion. This noncovalent association between GARP and latent TGF-β is sufficient for GARP to outcompete LTBPs [73, 89]. It seems that the disulfide-linkage is unnecessary for binding between GARP and latent TGF-β. In fact, the disulfide-linkage is indispensable for the release of mature TGF-β because the mutant is unable to activate TGF-β [89]. The RGD motif in LAP is recognized by integrin α_v_β_6_ and α_v_β_8_ [90, 91], thus inducing the release of active TGF-β from latent TGF-β/GARP complex [92, 93]. Despite soluble GARP (sGARP) forms an complex with latent TGF-β, sGARP is unable to support α_v_β_6_ or α_v_β_8_ mediated TGF-β activation [73]. This suggests that membrane form of GARP not sGARP is necessary for TGF-β activation. Overall, membrane GARP is not enough to release TGF-β. The recognition of RGD motif by integrin and the linkage between surface GARP and latent TGF-β are all required to TGF-β activation [73].

#### TGF-β participates in tregs function and homeostasis

Mature TGF-β released from Tregs can act on target cells in paracrine manner and plays a critical role for function of Tregs. TGF-β inhibits Teffs proliferation and cytokines production [94]. It also suppresses the differentiation of Teffs into Th1 and Th17 cells [95]. Besides, Tregs suppress anti-tumor immunoresponses and CD8^+^ T cells proliferation *via* TGF-β. TGF-β receptor negative CD8^+^ T cells abrogate this anti-tumor immunoresponses [96, 97]. Because TGF-β release process from Tregs is mediated by GARP, GARP may play a role in Tregs function through TGF-β.

Interestingly, studies have certificated that mTGF-β is involved in cell-contact dependent immunosuppression of Tregs [98–100]. Feline Tregs from immunodeficiency virus infection inhibit the proliferation and the cytokines production of Th cells. These Tregs are able to convert Th cells into iTregs through GARP-mTGF-β [101, 102]. Moreover, GARP-mTGF-β complex rather than secreted TGF-β from Tregs has the ability to induce Th17 differentiation. On the other hand, GARP can discriminate CD25^+^ T cells that contain high levels of IL-17 secreting cells [81]. Two anti-GARP monoclonal antibodies recognize a conformational epitope within GARP-mTGF-β complex and block the production of active TGF-β from human Tregs. The two antibodies can effectively inhibit the immunosuppressive activity of human Tregs in a model of xenogeneic graft-*versus*- host disease in NSG mouse [103]. But the function of GARP-mTGF-β complex is still unclear and further explorations are needed.

TGF-β from Tregs is also able to act on Tregs themselves, maintaining the function and homeostasis of Tregs [104]. TGF-β specific transcriptional prolifing and phosphorylated Smads are detected in human and mice Tregs [105]. Both Smad2 and Smad3 have the capacity to induce the expression of Foxp3 [106–108]. Besides, the interaction of Smad3 and CNS1 (an enhancer region of Foxp3 gene) is essential for maintaining Tregs number and homeostasis *in vivo* [39, 108]. Moreover, Tregs of TGF-β receptor-deficient has increaesd apoptosis [104, 109]. These uncover that TGF-β from Tregs forms the autocrine and positive TGF-β loop, maintaining the function and homeostasis of Tregs.

## IMPLICATIONS IN CANCER IMMUNOTHERAPY

In mice with colitis-associated colon cancer, Tregs from tumor express higher GARP and CTLA-4. Theses Tregs possess strong suppressive activity. The frequency and cytotoxic activity of CD8^+^ T cells are enhanced by the transient ablation of GARP^+^CTLA-4^+^ Tregs, thus attenuating tumor growth [110]. In head and neck squamous cell carcinoma patients, the frequency of highly suppressive Tregs with upregulated GARP and LAP is increased by adjuvant chemoradiotherapy (CRT). These Tregs from CRT patients are resistant to cisplatin and activation induced cell death. Additionally, these Tregs participate in suppressing anti-tumor immunoresponses and recurrence of cancer [111]. Moreover, the frequency of GARP^+^Foxp3^+^ Tregs is highly elevated in peripheral blood of patients with advanced hepatocellular carcinoma. The depletion of GARP^+^ Tregs in combination with CTLA-4 restores CD8^+^ T cell-dependent granzyme production [112]. CD8^+^ T cells expressing high levels of granzyme B are associated with prolonged progression free survival after combination of rituximab and chemotherapy in follicular lymphoma patients [113]. Thus, GARP together with other molecules in depleting Tregs or attenuating their suppressive activity may represent a new target in cancer immunotherapy.

## CONCLUSIONS

The discovery of surface GARP on Tregs sets a new stage in elucidating functions and mechanism of Tregs. GARP provides a regulatory network between Tregs and its targets including Tregs themselves. GARP^+^ Tregs may be used as targets especially in tumor immunotherapy. We should notice that the regulaory function mediated by GARP-TGF-β pathway in Tregs is not the whole story but just a part. Many other membrane molecules such as CTLA-4 and other checkpoints are also reported to mediate the suppressive function of Tregs. However, GARP-deficient Tregs are present in normal numbers in peripheral tissues of mice. Besides, these GARP-deficient Tregs develop normally and are as effective as Tregs from WT mice in suppressing CD4+CD25- responder cell proliferation [81]. It indicates that GARP is not absolutely required for the suppressive function of Tregs. But until now our understanding of GARP is very little and not very clear. The thorough and clear elucidation is a vital and difficult challenge in the future. Further studies may make it become a crucial molecule for Tregs even for the isolation of Tregs.
